# Human Brucellosis, Inner Mongolia, China

**DOI:** 10.3201/eid1612.091081

**Published:** 2010-12

**Authors:** Wen-Yi Zhang, Wei-Dong Guo, Shan-Hua Sun, Jia-Fu Jiang, Hai-Long Sun, Shen-Long Li, Wei Liu, Wu-Chun Cao

**Affiliations:** Author affiliations: People's Liberation Army Institute of Disease Control and Prevention, Beijing, People's Republic of China (W.-Y. Zhang, H.-L. Sun, S.-L. Li);; Beijing Institute of Microbiology and Epidemiology, Beijing (W.-Y. Zhang, J.-F. Jiang, W. Liu, W.-C. Cao);; Inner Mongolia Center for Disease Control and Prevention, Hohhot, People's Republic of China (W.-D. Guo);; Chinese Center for Disease Control and Prevention, Beijing (S.-H. Sun)

**Keywords:** Brucellosis, Brucella spp., China, bacteria, zoonoses, letter

**To the Editor:** Brucellosis is one of the most common zoonotic diseases worldwide (*1*). The disease is caused by *Brucella* spp. and mainly transmitted from its animal reservoirs to humans by direct contact with infected animals or through the ingestion of raw milk or unpasteurized cheese (*2*). Human brucellosis has a wide spectrum of clinical manifestations, which can vary from subclinical infection with seroconversion to a full-blown clinical picture of fever; osteoarticular involvement; sweating; constitutional symptoms; and hepatic, cardiac, central nervous system, or ocular involvement (*2**–**4*). Although controlled in many industrialized countries, the disease remains endemic to many parts of the world, including Spain, Latin America, the Middle East, parts of Africa, and Asia (*5*). In the People’s Republic of China, human brucellosis was highly endemic from the mid-1950s well into the 1970s, but then incidence decreased until the mid-1990s. However, incidence has increased sharply in China since 1995 (*6*), and the Inner Mongolia Autonomous Region is the most severe endemic focus; most reports of the disease occurred during 1999–2008. National and local public health authorities are concerned about the increasing incidence of the disease in this province. Here we report the epidemic characteristics that existed in this region during 1999–2008.

Human brucellosis is a reportable disease in China; suspected or confirmed cases must be reported to local and provincial Centers for Disease Control and Prevention (CDC) and then to Chinese CDC (CCDC) through the National Notifiable Disease Surveillance System. To meet case definitions, disease in persons must be accompanied by clinical signs and must be confirmed by serologic tests or isolation in accordance with the case definition of the World Health Organization (*1**,**7*).

We obtained the National Notifiable Disease Surveillance System data that were confirmed by the Chinese CDC from Inner Mongolia CDC. A total of 43,623 cases were reported during 1999–2008, of which 70.7% occurred in male patients; the difference in incidence between sexes was significant by χ^2^ test (χ^2^ = 581.9, p<0.00001). A total of 28,237 (64.7%) reported cases occurred in persons 30–59 years of age, male (70.2%) and female (29.8%). However, 658 patients (396 boys) were <10 years of age, and 497 patients (333 men) were >70 years of age. The number of cases peaked in 2008, with 7,645 and 3,460 cases in male and female patients, respectively. The epidemic peaked in March–August, with 74.8% reported cases during the study period. The number of reported cases in 2008 was 25.6× the number reported in 1999. The highest proportion of cases (55.9%) occurred among persons engaged in agricultural activities (planting, animal husbandry) in rural areas; the next highest proportion was in shepherds (29.2%), who depend only on their herds to satisfy their nutritional needs. The number of cases sharply increased from 37 and 16 in 2001 to 315 and 308 in 2008 among housekeepers and students, respectively. In this province, *B. melitensis* was the most common pathogen, although *B. abortus* prevailed in certain regions. During our epidemiologic investigation, the number of agriculture workers who were inexperienced in animal husbandry increased suddenly and quickly; thus the trade and transportation of unquarantined and unvaccinated animals rose sharply. This situation most likely led to easier transmission to humans by direct contact with infected animals than had occurred previously. The results of our investigation indicate that the main risk factors associated with this outbreak were occupation (agriculture worker, shepherd, butcher, slaughterhouse worker, and cattle dealer) and risky practices (handling of ruminant abortions, skinning of stillborn lambs and kids, and crushing the umbilical cord of newborn lambs and kids with teeth) and certain dietary preferences (consuming unpasteurized and unboiled milk and fresh cheese) (W. Guo, pers. comm.).

Our results show that the annual incidence of the disease varied greatly from 0 to 818.52/100,000 at county levels during the study period (Figure). The largest incidence of the disease occurred in Abaga County in the center of Inner Mongolia. The spatial distribution of the disease clustered in the northeastern (Hulunbeir) and central (Xilinguole) districts. Hence, future public health planning and resource allocation should focus on Hulunbeir and Xilinguole, and active surveillance should be strengthened in these high-risk districts.

**Figure:**
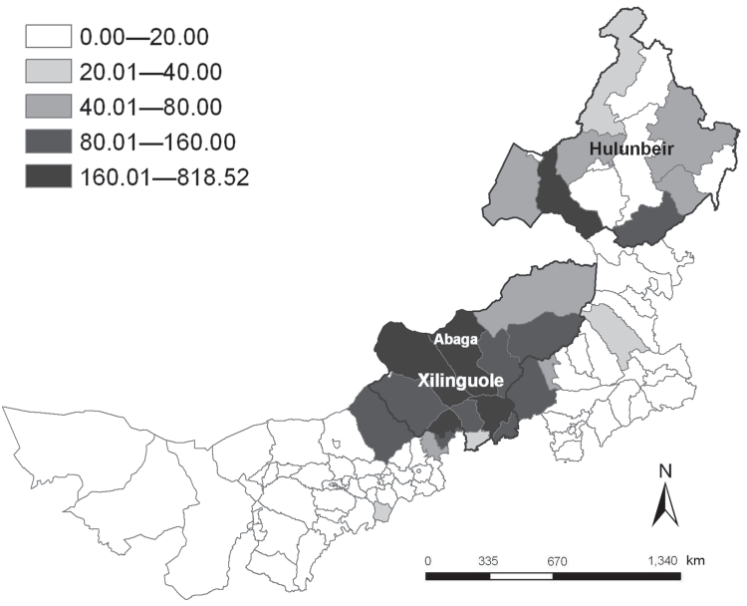
Annualized average incidence of human brucellosis, Inner Mongolia Autonomous Region, People’s Republic of China, 1999–2008.

We report the epidemic features of human brucellosis in a province in China. This information will be helpful to establish strategies for prevention, surveillance, and management of human brucellosis in China and in other countries where the disease is endemic.
